# Antimicrobial activities of the methanol extract and compounds from the twigs of *Dorstenia mannii* (Moraceae)

**DOI:** 10.1186/1472-6882-12-83

**Published:** 2012-06-29

**Authors:** Armelle T Mbaveng, Victor Kuete, Bathelemy Ngameni, Veronique P Beng, Bonaventure T Ngadjui, Jacobus J Marion Meyer, Namrita Lall

**Affiliations:** 1 Department of Biochemistry, Faculty of Science, University of Dschang, P.O. Box 67, Dschang, Cameroon; 2 Department of Biochemistry, Faculty of Science, University of Yaounde I, P.O. Box 812, Yaounde, Cameroon; 3 Department of Pharmacy and Traditional Pharmacopoeia, Faculty of Medicine and Biomedical Science, University of Yaoundé I, Yaounde, Cameroon; 4 Department of Organic Chemistry, Faculty of Science, University of Yaoundé I, Yaounde, Cameroon; 5 Department of Plant Science, Faculty of Agricultural and Biological Science, Pretoria, 0002, South Africa

## Abstract

**Background:**

*Dorstenia mannii* (Moraceae) is a medicinal herb used traditionally for the treatment of many diseases. In the present study, the methanol extract of *D. mannii* and nine of its isolated compounds*,* namely dorsmanin A (**1**), B (**2**), C (**3**), D (**4**), E (**6**), F (**7**), G (**8**) dorsmanin I (**9**) and 6,8-diprenyleriodictyol (**5**), were tested for their antimicrobial activities against yeast, Mycobacteria and Gram-negative bacteria.

**Methods:**

The microplate alamar blue assay (MABA) and the broth microdilution method were used to determine the minimal inhibitory concentration (MIC) and minimal microbicidal concentration (MMC) of the above extract and compounds on a panel of bacterial species.

**Results:**

The results of the MIC determinations demonstrated that the methanol extract as well as compounds **3** and **8** were able to prevent the growth of all the fourteen studied microorganisms within the concentration range of 4 to 1024 μg/ml. The lowest MIC value for the methanol extract (64 μg/ml) was obtained on *Candida albicans*. The lowest value for individual compounds (4 μg/ml) was recorded with compounds **3** on *Pseudomonas aeruginosa* PA01 and **7** on *Eschericia coli ATCC* strain. The MIC values recorded with compounds **3** on *P. aeruginosa* PA01, **6** on *C. albicans,***7** on *P. aeruginosa* PA01 and *K. pneumoniae* ATCC strain and *C. albicans,*and **8** on *P. aeruginosa* PA01, PA124, *P. stuartii*, *M. tuberculosis* MTCS1 were lower than or equal to those of the reference drugs. MMC values not greater than 1024 μg/ml were recorded on all studied microorganisms with compounds **3** and **8**.

**Conclusion:**

The overall results of the present investigation provided evidence that the crude extract of *D. mannii* as well as some of its compounds such compounds **3** and **8** could be a potential source of natural antimicrobial products.

## Background

Many plant species of the genus *Dorstenia* (Moraceae) are used for medicinal purposes in Africa, Middle East, Central and South America. African *Dorstenia* species has yielded a variety of mono-, di-, and triprenylated and also mono- and digeranylated flavonoids with interesting pharmacological properties [1-4]. *Dorstenia mannii* Hook f. (Moraceae) is a perennial herb growing in the tropical rain forest of West Africa [5]. A decoction of the leaves is used for the treatment of many diseases, but mainly for rheumatism and stomach disorders [6]. There are few pharmacological studies reported on *D. mannii*. However, prenylated flavonoids isolated from *D. mannii* such as 6,8-diprenyleriodictyol (**5**), dorsmanin C (**3**) and dorsmanin F (**7**) were found to be potent scavengers of the stable free radical 1,1-diphenyl-2-picrylhydrazyl [7]. Compounds **3, 5** and **7** also inhibited Cu^2+^-mediated oxidation of human low density lipoprotein [7]. In our continuous search of bioactive compounds from the genus *Dorstenia,* the present work was designed to evaluate the antimicrobial potency of the methanol extract and compounds isolated from *D. mannii.*

## Methods

### Plant material and extraction

The twigs of *Dorstenia mannii* Hook. f. were collected at Nkoljobe mountain, Yaounde, Center region of Cameroon in March 2008. The plant was identified by Mr. Victor Nana of the National herbarium (Yaoundé, Cameroon) where a voucher specimen was deposited under the reference number 2135/HNC.

The air dried and powdered twigs (1 kg) were extracted with methanol (MeOH) for 48 h at room temperature. The extract was then concentrated under reduced pressure to give 185 g of a brown residue that constituted the crude extract (DMT).

### Chemicals for antimicrobial assay

**Figure 1:**
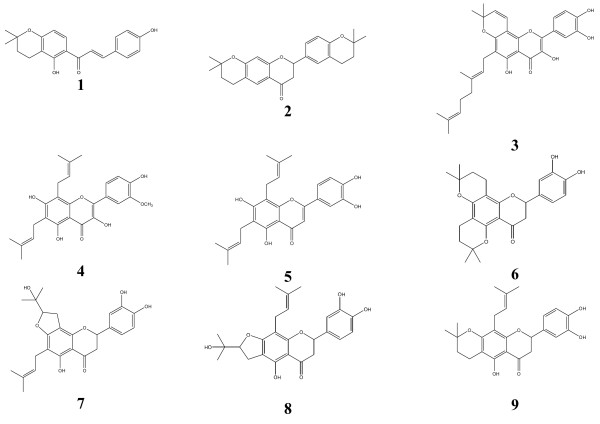
**Chemical structures of the compounds isolated from the twigs of*Dortenia mannii*.** Dorsmanins A(**1**), B(**2**), C(**3**), D(**4**), E (**6**), F (**7**), G (**8**), I (**9**) and 6,8 diprenyleriodictyol (**5**).

Chloramphenicol (Sigma-Aldrich, St. Quentin Fallavier, France) and Nystatin (Sigma-Aldrich) were used as reference antibiotics (RA) respectively against bacteria and *Candida albicans*. *p*-Iodonitrotetrazolium chloride (INT, Sigma-Aldrich) was used as microbial growth indicator [8,9]. Ciprofloxacin and isoniazid (INH) (Sigma) were used as reference antibiotics (RA) for *M. smegmatis* and *M. tuberculosis* respectively. The isolation and identification of compounds **1** to **9** from DMT were conducted as previously described [10-12]. The chemical structures of the isolated compounds are illustrated in Figure 1.

### Antimicrobial assays

#### Microbial strains and culture media

The studied microorganisms included strains of *Providencia stuartii, Pseudomonas aeruginosa, Klebsiella pneumoniae, Enterobacter aerogenes, Escherichia coli, Candida albicans* four *Mycobacteria* namely *M. smegmatis*, drug-susceptible strain of *M. tuberculosis* H37Rv obtained from the American Type Culture Collection, and two clinical strains of *M. tuberculosis* MTCS1, MTCS2. *M. smegmatis* was cultured on Middlebrook 7 H11 agar and allowed to grow for 24 h. *M. tuberculosis* was plated on Löwenstein–Jensen medium and allowed to grow for 3–4 weeks at 37°C. Middlebrook 7 H9 broth was used to determine the MIC and MMC values of the test samples on *M. smegmatis* and *M. tuberculosis*. Nutrient Agar and Sabouraud Glucose Agar were used for the activation of Gram-negative bacteria and fungi respectively [13]. The clinical strains used in this work are our Laboratory collection previously obtained from Yaoundé General Hospital (Cameroon), and from the Mediterranean University (Marseille, France).

#### INT colorimetric assay for MIC and MMC determinations

The MIC determinations on *M. smegmatis,* fungi, and Gram-negative bacteria were conducted using rapid INT colorimetric assay according to previously described methods [8,9] with some modifications. The test samples and RA were first of all dissolved in DMSO/MHB or DMSO/7 H9 broth. The final concentration of DMSO was lower than 2.5% and does not affect the microbial growth [14]. The solution obtained was then added to 7 H9 broth (*M. smegmatis*) or MHB (other organisms), and serially diluted two fold (in a 96- wells microplate). 100 μl of inoculum 1.5 x 10^6^ CFU/ml prepared in appropriate broth was then added [15]. The plates were covered with a sterile plate sealer, then agitated to mix the contents of the wells using a plate shaker and incubated at 37°C for 18 h. The assay was repeated thrice. Wells containing adequate broth, 100 μl of inoculum and DMSO to a final concentration of 2.5% served as negative control. The MIC of samples was detected after 18 h incubation at 37°C, following addition (40 μl) of 0.2 mg/ml *p*-iodonitrotetrazolium chloride (INT) and incubation at 37°C for 30 minutes. Viable bacteria reduced the yellow dye to a pink. MIC was defined as the sample concentration that prevented this change and exhibited complete inhibition of microbial growth. The MMC was determined by adding 50 μl aliquots of the preparations, which did not show any growth after incubation during MIC assays, to 150 μl of adequate broth. These preparations were incubated at 37°C for 48 h. The MMC was regarded as the lowest concentration of extract, which did not produce a color change after addition of INT as mentioned above [14,16].

#### Microplate Alamar Blue assay against M. tuberculosis

The activity of all samples against *M. tuberculosis* strains was tested using the MABA [17]. Briefly, each of the above *M. tuberculosis* strains was cultured at 37°C in Middlebrook 7 H9 broth supplemented with 0.2% glycerol and 10% Oleic Acid–Albumin–Dextrose–Catalase (Sigma) until logarithmic growth was reached. About 6x10^6^ CFU/ml inoculum of *M. tuberculosis* was then added to the two fold serially diluted samples. The final concentration of DMSO in all assays was 2.5% or less and this dilution also served as solvent control. The samples were assayed in triplicate. All tests were carried out in sterile flat-bottomed 96-well microplates. Each microplate was incubated for 5 days at 37°C in a 5% CO_2_ atmosphere in a sealed plastic CO_2_-permeable bag. After 5 days of incubation, 32 μl of a mixture of freshly prepared Alamar Blue solution and 20% sterile Tween-80 (Sigma) 1:1 v/v were added to one growth-control well. The microplates were incubated again at 37°C for 24 h. If a color shift from blue to pink was observed in the growth-control sample, 32 μl of alamar blue solution was added to each of the remaining wells, and the microplate was further incubated for 24 h. A well-defined pink color was interpreted as positive bacterial growth, whereas a blue color indicated an absence of growth. The MIC corresponded to the greatest dilution of sample extract in which the color shift from blue to pink was not observed.

Samples with recorded MIC values following MABA were assayed for their mycobactericidal effect [17]. Briefly, 5 μl of the undeveloped mycobacterial suspensions were transferred from the former to a new microplate that contained 195 μl of fresh culture medium per well. Three wells were inoculated with 100 μl of fresh inoculum as for MABA and three more wells were incubated with 200 μl of culture medium only, as negative controls. The microplates were incubated and developed with alamar blue as for MABA. The MMC corresponded to the minimum sample concentration that did not cause a color shift in cultures that were re-incubated in fresh medium.

## Results and discussion

The tested compounds were isolated from DMT and identified as previously described as dorsmanin A (**1**), B (**2**), C (**3**), D (**4**) and 6,8-diprenyleriodictyol (**5**) [10], dorsmanin E (**6**), F (**7**), G (**8**) [11] and dorsmanin I (**9**) [12]. These compounds together with the crude methanol extract were tested for their antimicrobial activities against bacteria and yeasts and the results are reported in Tables 1 and 2.

**Table 1:** Minimal inhibitory concentrations (MIC in μg/ml) of the studied samples and reference antibiotics against the tested microorganisms

Tested samples*	**Microorganisms, strains and MIC (μg/ml)**													
	***E. coli***		***P. aeruginosa***		***K. pneumoniae***		***E.aerogenes***		***P. stuartii***	***C. albicans***	***M. smegmatis***	***M. tuberculosis***		
	***ATCC10536***	***AG100***	***PA01***	***PA124***	***ATCC11296***	***KP55***	***ATCC13048***	***CM64***	***NAE16***	***ATCC 9002***	***ATCC 700084***	***ATCC 27294***	***MTCS1***	***MTCS2***
DMT	256	512	512	1024	128	512	128	512	128	64	128	128	1024	512
**1**	512	-	-	-	-	-	128	-	128	-	-	NT	NT	NT
**2**	128	128	-	-	-	-	-	-	1024	-	512	512	-	-
**3**	64	64	4	64	128	64	32	64	16	64	64	32	128	32
**4**	-	-	128	-	512	-	-	-	1024	-	-	NT	NT	NT
**5**	512	-	-	-	1024	-	32	-	128	32	-	NT	NT	NT
**6**	512	128	512	-	128	-	16	-	256	8	-	NT	NT	NT
**7**	4	256	32	-	8	64	16	-	64	16	128	128	256	128
**8**	16	128	8	32	128	32	64	32	32	128	64	64	64	64
**9**	-	-	-	-	-	-	256	-	256	32	128	256	-	512
RA^b^	2	8	64	32	8	4	8	4	32	16	0.5	0.5	64	2

^a^Tested samples [DMT: methanol extract from the twigs of *Dorstenia mannii*; dorsmanins A(**1**), B(**2**), C(**3**), D(**4**) E(**6**), F(**7**), G(**8**), I (**9**) and 6,8 diprenyleriodictyol (**5**); ^b^RA : reference antibiotics were chloramphenicol for bacteria, nystatin for *C. albicans,* ciprofloxacin for *M. smegmatis,* isoniazid for *M.tuberculosis*; (−): MIC > 1024 μg/ml

**Table 2:** Minimal microbicidal concentrations (MMC in μg/ml) of the studied samples and reference antibiotics against the tested microorganisms

Tested samples*	**Microorganisms, strains and MMC (μg/ml)**													
	***E. coli***		***P. aeruginosa***		***K. pneumoniae***		***E.aerogenes***		***P. stuartii***	***C. albicans***	***M. smegmatis***	***M. tuberculosis***		
	***ATCC10536***	***AG100***	***PA01***	***PA124***	***ATCC11296***	***KP55***	***ATCC13048***	***CM64***	***NAE16***	***ATCC 9002***	***ATCC 700084***	***ATCC 27294***	***MTCS1***	***MTCS2***
DMT	512	>1024	1024	>1024	512	1024	512	>1024	256	128	256	256	>1024	>1024
**1**	>1024	ND	ND	ND	ND	ND	1024	ND	1024	ND	ND	NT	NT	NT
**2**	1024	1024	ND	ND	ND	ND	ND	ND	>1024	ND	512	>1024	ND	ND
**3**	128	128	8	128	256	128	64	128	64	128	128	64	256	64
**4**	ND	ND	1024	ND	>1024	ND	ND	ND	>1024	ND	ND	NT	NT	NT
**5**	>1024	ND	ND	ND	>1024	ND	128	ND	1024	64	ND	NT	NT	NT
**6**	>1024	>1024	>1024	ND	256	ND	64	ND	>1024	16	ND	NT	NT	NT
**7**	8	512	64	ND	16	256	32	ND	256	32	256	256	1024	512
**8**	32	512	16	64	256	128	128	128	64	256	128	128	128	128
**9**	ND	ND	ND	ND	ND	ND	1024	ND	>1024	64	1024	512	ND	>1024
RA^b^	4	32	128	128	32	16	16	16	128	32	1	1	128	4

^a^Tested samples [DMT: methanol extract from the twigs of *Dorstenia mannii*; dorsmanins A(**1**), B(**2**), C(**3**), D(**4**) E(**6**), F(**7**), G(**8**), I (**9**) and 6,8 diprenyleriodictyol (**5**); ^b^RA : reference antibiotics were chloramphenicol for bacteria, nystatin for *C. albicans,* ciprofloxacin for *M. smegmatis,* isoniazid for *M.tuberculosis*; (−): MIC > 1024 μg/ml; (ND): not determined as MIC was >1024 μg/ml

The results of the MIC determinations (Table 1) demonstrated that the methanol extract as well as compounds **3** and **8** were able to prevent the growth of all the fourteen studied microorganisms, including mycobacteria, yeast and Gram-negative bacteria, within the concentration range of 4 to 1024 μg/ml. Other compounds showed selective activities, their inhibitory effects being noted on 12/14 (85.7%) studied pathogens for compound **7**, 7/14 (50%) for compound **6**, 6/14 (42.9%) for compounds **9**, 5/14 (35.7%) for compounds **2** and **5**, 3/14 (21.4%) for compounds **1** and **4**. The lowest MIC value for the methanol extract (64 μg/ml) was obtained on *C. albicans*. The lowest value for individual compounds (4 μg/ml) was recorded with compounds **3** on *P. aeruginosa* PA01 and **7** on *E. coli ATCC* strain. The corresponding values for the RA ranged from 0.5 to 64 μg/ml, *P. aeruginosa* PA01 and *M. tubercolosis* MTCS1 being the least sensitive. Results of MMC determinations (Table 2) also showed good activities for some of the tested samples such as compounds **3** and **8**. MMC values not greater than 1024 μg/ml were recorded on all studied microorganisms with compounds **3** and **8**, on 12/14 (85.7%) studied organisms for compound **7**, 9/14 (64.3%) for the crude extract, 4/14 (28.6%) for compound **9**, 3/14 (21.4%) for compounds **5** and **6**, 2/14 (14.3%) for compounds **1** and **2**, 1/14 (7.1%) for compound **4**.

The compounds isolated from *D. mannii* and tested herein were all flavonoids. This class of compounds is very common in the genus *Dorstenia*[10-12] and their antimicrobial activities were also reported [2-4]. In the present work, broad spectrum of antimicrobial activities was recorded with the crude extract and compounds from *D. mannii.* Phytochemicals are routinely classified as antimicrobials on the basis of susceptibility tests that produce MIC in the range of 100 to 1000 μg/ml [18]. Activity is considered to be significant if MIC values are below 100 μg/ml for crude extract and moderate when the MIC values vary from 100 to 625 μg/ml [19,20]. Therefore, the activity recorded with the crude extract on *C. albicans* can be considered as important. Also, compounds with significant activities (MIC < 10 μg/ml) on at least one of the studied organisms include **3, 6, 7** and **8**. The MIC values recorded with compounds **3** on *P. aeruginosa* PA01, **6** on *C. albicans,***7** on *P. aeruginosa* PA01 and *K. pneumoniae* ATCC strain, and *C. albicans,***8** on *P. aeruginosa* PA01, PA124, *P. stuartiiM. tuberculosis* MTCS1 were lower than or equal to those of the reference drugs, highlighting their interesting activities. This observation is in consistence with previous work on flavonoids isolated from the genus *Dorstenia.* In fact, isobavachalcone, 4-hydroxylolonchocarpin, kanzonol C, stipulin, and many other flavonoids isolated from this genus were reported for their good antimicrobial potencies, with MIC values below 10 μg/ml on several tested microorganisms [2-4,21]. A Keen look at the MMC values indicates that most of them are not more than fourfold their corresponding MICs. This proves that the killing effects of many tested samples could be expected on the sensitive strains [22]. The continuous emergence of multidrug-resistant (MDR) bacteria drastically reduces the efficacy of our antibiotic armory and, consequently, increases the frequency of therapeutic failure [23]. MDR Enterobacteriaceae, including *K. pneumoniae, E. aerogenes and E. coli* have also been classified as antimicrobial-resistant organisms of concern in healthcare facilities [24]. Besides, *K. pneumoniae* KP55 tested herein was reported to be resistant to most of the commonly used antibiotics, showing high levels of resistance to ampicillin, ceftazidime, and aztreonam with MIC values up to 512 μg/ml [25]. In addition *Pseudomonas aeruginosa* has emerged as one of the most problematic Gram-negative pathogens, with the alarmingly high antibiotics resistance rates [26]. The good activities of compounds **3** and **8** on most of the tested strains belonging to MDR phenotypes such as *E. coli* AG100, *P. aeruginosa* PA124, *E. aerogenes* CM64, *K. pneumoniae* KP55 as observed herein reinforce the hypothesis that these compounds are natural products with interesting antimicrobial potencies.

Tuberculosis (TB) is widely expanded in poor countries with the highest incidence (more than 80% of cases) occurring in Asia and Africa [27]. Annual incidence of TB (over 600 cases per 100 000) has been reported in many sub-Saharan African countries [28]. In this work, only compounds with inhibitory activity on *M. smegmatis* were tested on *M. tuberculosis*. However, it has been demonstrated that the sensitivity of *M. tuberculosis* is closer to that of *M. smegmatis*, a non pathogenic microorganism [29]. Therefore, this microorganism can be used for a preliminary study to select samples with potential activity against *M. tuberculosis*[29]. Hence, the results obtained herein are in accordance with such recommendation.

To the best of our knowledge, the antimicrobial activity of *D. mannii* as well as that of the isolated compounds is being reported for the first time. However 6,8-diprenyleridictyol (**5**) an,dorsmanin F (**7**) were reported for their antitrichomonal activities [30].

## Conclusion

The data reported herein are very important, taking into account the medical importance of the studied microorganisms. Hence, the overall results of the present investigation provide evidence that the crude extract of *D. mannii* as well as some of its compounds such as compounds **3** and **8** could be considered as interesting natural antimicrobial products.

## Competing interests

The authors declare that they have no competing interests.

## Authors’ contributions

ATM, VK and BN carried out the study; ATM and VK wrote the manuscript; VK, BTN, VPB, JJMM and NL supervised the work. All authors read and approved the final manuscript.

## Pre-publication history

The pre-publication history for this paper can be accessed here:

http://www.biomedcentral.com/1472-6882/12/83/prepub
